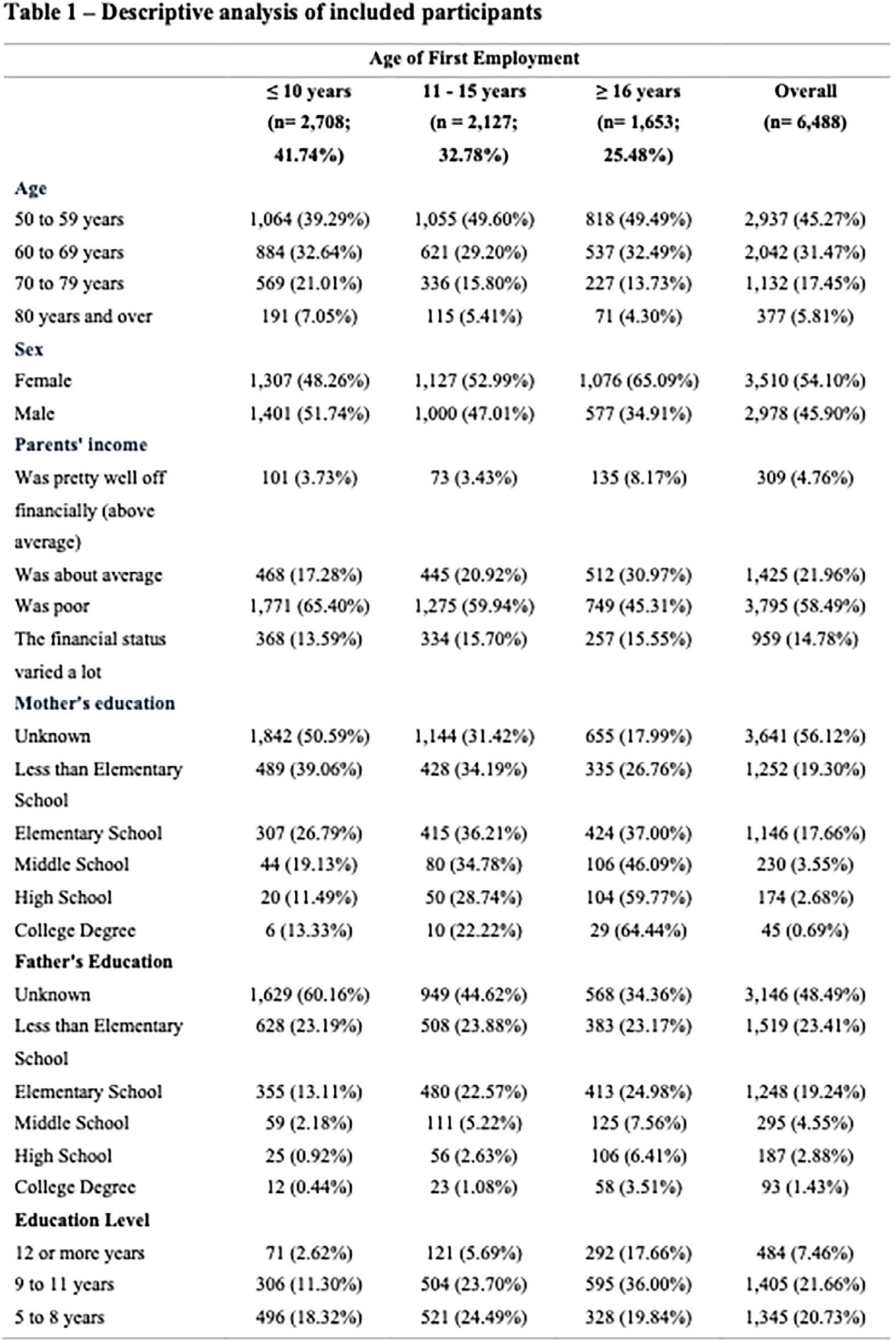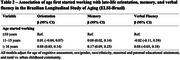# Early Work Initiation and Cognitive Function Among Brazilian Older Adults: Findings from the ELSI‐Brazil Study

**DOI:** 10.1002/alz70860_103283

**Published:** 2025-12-23

**Authors:** Bruna Fogaça, Greta Fehlan, Joya Deb Lucky, Kazi Sabrina Haq, Ione Jayce Ceola Schneider, Rachel Peterson

**Affiliations:** ^1^ University of Montana, Missoula, MT, USA; ^2^ Universidade Federal de Santa Catarina, Florianópolis, SC, Brazil

## Abstract

**Background:**

Economic hardship has led many Brazilians to enter the workforce prior to age 16. While early employment often limits formal education, its direct impact on late‐life cognition remains unclear. Employment may also provide cognitive stimulation that benefits late‐life cognition. We examined associations between age of first employment and late‐life cognitive function in a national cohort of Brazilians.

**Method:**

In the Brazilian Longitudinal Study of Aging (ELSI‐Br), temporal orientation, memory, and verbal fluency were assessed. Orientation was modeled as a raw score (0–4) due to a ceiling effect in the measure (strong left‐skewed distribution). Memory and verbal fluency z‐scores were standardized by age and sex by subtracting the mean and dividing by the standard deviation within each age and sex group of the full ELSI‐Br sample. Age at work initiation was categorized as ≤10 years, 11–15 years, and ≥16 years. Linear regression models estimated associations between early work initiation and cognitive scores, adjusting for race, rural residence, and both mother's and father's education.

**Result:**

Among complete cases (*n* = 6,488), 41.74% started working at or before age 10, 32.78% between 11–15 years, and 25.48% at age 16 or older (Table 1). In fully adjusted linear models, orientation scores trended higher among those who started working at ages 11‐15 (β=0.01 [95% CI=‐0.04, 0.07]) and were significantly higher among those who started working after age 16 (β=0.08 [95% CI=0.03, 0.14]) compared to those who initiated employment at age 10 or younger (ref.). Memory scores were significantly higher among those who initiated employment at ages 11‐15 (β=0.8 [95% CI=0.02, 0.14) and after age 16 (β=0.17 [95% CI=0.09, 0.25]) compared to the reference. Verbal fluency scores trended slightly lower among those who initiated employment at ages 11‐15 (β=‐0.02 [95% CI = ‐0.11, 0.58]) and trended higher among those who started work after age 16 (β=0.08 [95% CI = ‐0.03, 0.18]) compared to the reference.

**Conclusion:**

These findings highlight the association between early workforce entry and late‐life cognition and can inform policies promoting education and delayed employment in vulnerable populations. Future work will explore education and employment as mediators.